# HIF-1 Inhibitor YC-1 Reverses the Acquired Resistance of EGFR-Mutant HCC827 Cell Line with MET Amplification to Gefitinib

**DOI:** 10.1155/2021/6633867

**Published:** 2021-03-03

**Authors:** Qian Jin, Jisheng Zheng, Ming Chen, Na Jiang, Xianrong Xu, Feihua Huang

**Affiliations:** Department of Respiratory Medicine, Tongde Hospital of Zhejiang Province, Hangzhou, Zhejiang 310012, China

## Abstract

**Background:**

Acquired resistance occurred in the majority of nonsmall cell lung cancer (NSCLC) patients receiving epidermal growth factor receptor-tyrosine kinase inhibitors (EGFR-TKIs) therapy, and this may be related to the activation of the HIF-1 pathway. Therefore, we examined the influence of the hypoxia-inducible factor-1 (HIF-1) pathway inhibition on the sensitivity of HCC827 gefitinib-resistant (HCC827 GR) cells with MET amplification to gefitinib.

**Methods:**

We established HCC827 GR cell line with MET amplification and set four groups with different treatment. An MTT assay, a colony formation analysis, and a wound healing assay were performed to determine the sensitivity change of HCC827 GR cells after different treatments. HIF-1*α*, p-EGFR, and p-Met levels were detected with western blot. Correlations among HIF-1*α*, p-EGFR, and p-Met levels of HCC827 GR cells with different treatments were analyzed with Pearson's correlation analysis.

**Results:**

HIF-1 inhibitor YC-1 enhanced the sensitivity of HCC827 GR cells to gefitinib. p-Met level was correlated with HIF-1*α* level, while there was no correlation between p-Met level and p-EGFR level.

**Conclusion:**

HIF-1 inhibitor YC-1 is able to reverse the acquired resistance of HCC827 GR to gefitinib, and the regulation of the HIF-1 pathway on MET may be one of the mechanisms.

## 1. Introduction

The acquired resistance of anticancer drugs is a major cause for therapeutic failure in nonsmall cell lung cancer (NSCLC) leading to tumor recurrence, progression, and poor prognosis [1]. For NSCLC patients with EGFR sensitive mutation, epidermal growth factor receptor-tyrosine kinase inhibitors (EGFR-TKIs) have been used clinically as the first-line treatment [2–4]. However, tumor progression inevitably occurred in the majority of NSCLC patients receiving EGFR-TKIs therapy despite the initial obvious and rapid effects of EGFR-TKIs [5]. Many mechanisms such as T790M mutation, human EGFR-2 amplification, and MET amplification may lead to acquired resistance of EGFR-TKIs [6, 7], but there must be many other mechanisms that need further researches.

Hypoxia is a remarkable characteristic of lung cancer [8]. Tumors in hypoxia condition are easier to have gene mutation, more resistant to antitumor therapy, more invasive, and more antiapoptotic [9]. Under hypoxia condition, the hypoxia-inducible factor 1 (HIF-1) signaling pathway is activated and plays an important role on the biological effects of hypoxia [8]. HIF-1 consists of a functional *α* submit and a *β* subunit [10]. In a previous study, the quantity of NSCLC stem cells which were resistant to EGFR-TKIs in EGFR mutant NSCLC was increased under hypoxia condition, and the HIF-1*α* level was elevated in acquired EGFR-TKI-resistant NSCLC cells [11, 12]. Therefore, we aim at the HIF-1 pathway as a potential target to affect the sensitivity of NSCLC cells to EGFR-TKIs.

In our previous published research, we used HIF-1 inhibitor and activator to regulate the activity of the HIF-1 pathway and found that HIF-1 inhibitor can enhance the sensitivity of HCC827 cells (EGFR-TKIs sensitive EGFR exon 19 mutant NSCLC cell line) to EGFR-TKIs [13]. In order to learn the effect of the HIF-1 pathway on EGFR-TKI acquired resistant NSCLC, we design the present research.

3-(5′-hydroxymethyl-2′-furyl)-1-benzylindazole (YC-1) is a kind of benzyl indazole by chemically synthetizing [14]. It had been found as a HIF-1 inhibitor without cytotoxicity [15]. For the present study, YC-1 and gefitinib were selected as HIF-1 inhibitor and EGFR-TKI, respectively. HCC827 gefitinib-resistant (HCC827 GR) cell line was selected as the acquired EGFR-TKI resistant NSCLC cell line. HCC827 GR is generated by exposing HCC827 cells to increasing concentrations of gefitinib, and MET amplification is the mechanism of its acquired resistance [7, 16, 17]. In EGFR-TKI-sensitive NSCLC cells, EGFR was able to regulate MET level through the HIF-1 pathway [18]. In acquired EGFR-TKI-resistance NSCLC cells with MET amplification, EGFR lost its regulation on MET, and whether the HIF-1 pathway remained the regulation on MET kept unclear [7]. In order to make clear the correlation between HIF-1 and MET, acquired gefitinib-resistant HCC827 GR cells with MET amplification was considered to be the ideal cell line for the present study.

Here, we researched whether HIF-1 inhibiting can reverse the acquired gefitinib resistance of HCC827 GR and detected the levels of p-EGFR, HIF-1*α*, and p-Met to explore whether the relative mechanism was associated with the regulation of HIF-1 on MET.

## 2. Materials and Methods

### 2.1. Reagents

Reagents and suppliers were as follows: Droplet Digital PCR QX200 system (Bio-Rad Laboratories Inc., Hercules, CA, USA); antibodies against phosphorylated hepatocyte growth factor receptor (p-Met), c-Met, phosphorylated EGFR (p-EGFR), and EGFR protein (Abcam, Cambridge, MA, USA); and QIAamp DNA Mini Kit (Qiagen, Hilden, Germany). Other reagents and suppliers had been described in our previous research of Jin et al. 2019 [13].

### 2.2. Establishment of HCC827 GR Cell Line

Human commercially available HCC827 cell line was bought from China Academy of Cell Resource Center, Shanghai Institutes for Biological Sciences as the parental cell. Cell viability of HCC827 in different gefitinib concentrations was measured by MTT assay. HCC827 was continuously exposed to gefitinib beginning at 0.001 *μ*M (equivalent to IC20 in parental HCC827) and increased in a stepwise manner to 1 *μ*M to generate a resistant cell line. The gefitinib concentrations was increased stepwise to 0.006 *μ*M, 0.05 *μ*M, 0.1 *μ*M, 0.5 *μ*M, and 1 *μ*M, equivalent to IC30, IC40, IC50, IC60, and IC70 in parental cells, respectively, until these cells recovered near-normal growth kinetics. The total procedure took 6 months. In order to confirm the successful establishment of HCC827 GR cell line, cell viability of HCC827 GR in different gefitinib concentrations was measured by MTT assay after culturing HCC827 GR in gefitinib-free condition for at least 4 days. At the same time, HCC827 cells were cultured in gefitinib-free condition concomitantly, and their sensitivity to gefitinib was not changed through the gefitinib sensitivity examination every 5 passages [19].

### 2.3. MET Amplification Detection

In the process of HCC827 GR cell line established, MET levels from parental HCC827 cell to HCC827 GR cell (the gefitinib concentration was increased gradually from 0 *μ*M to 1 *μ*M) were detected. MET levels of HCC827 GR cell with different treatment were detected too. Cells were collected and washed with PBS for 2 times. DNA was abstracted and purified with QIAamp DNA Mini Kit. MET amplifications were analyzed with ddPCR copy number variation (CNV) assay. QX200 ddPCR system was used to perform ddPCR. All procedures were performed according to instructions.

We followed the methods of Jin et al. 2019 [13] for the method of cell culture, medication treatment of YC-1, western blot assay, MTT assay, colony formation assay, cell migration assay, and statistical analyses. Concrete contents were described in supplementary material (available here)

## 3. Result

### 3.1. HCC827 GR Cell Line Was Established

**Figure 1:**
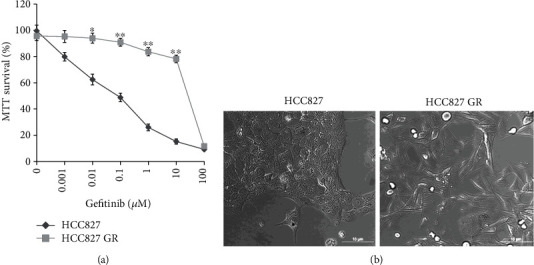
HCC827 GR cell line was established. (a) Gefitinib sensitivity between parental HCC827 cells and HCC827 GR cells was compared. Cell viability was measured by MTT assay. Data are presented as mean ± standard deviation from three independent experiments. ^∗^*P* < 0.05 and ^∗∗^*P* < 0.01 for HCC827 GR cells versus parental cells. (b) Morphological changes from parental HCC827 cells to HCC827 GR cells. Original magnification, ×200.

The parental cell HCC827 was continuously exposed to gefitinib beginning at 0.001 *μ*M and increased in a stepwise manner to 1 *μ*M. Finally, the HCC827 GR cell line was established as shown in Figure 1(a). Gefitinib had less effect on HCC827 GR cells than that on HCC827 cells. The IC50 of gefitinib on HCC827 GR cells and HCC827 cells was 26.53 ± 0.96 *μ*M and 0.08 ± 0.02 *μ*M. Moreover, the morphology of HCC827 GR cells were more elongated than their parental HCC827 cells (Figure 1(b)).

### 3.2. YC-1 Enhances the Sensitivity of HCC827 GR Cells to Gefitinib

**Figure 2:**
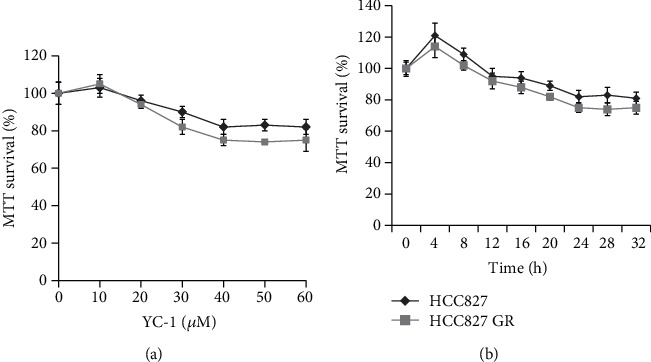
Effect of YC-1 on cell viability of parental HCC827 cells and HCC827 GR cells. (a) Cells were exposed to 10, 20, 30, 40, 50, and 60 *μ*M YC-1 and 0.066% DMSO for 24 h. YC-1 concentrations higher than 40 *μ*M were not able to further inhibit the viability of HCC827 and HCC827GR cells. (b) Cells were treated with 40 *μ*M YC-1 for different incubation times, and an increase of the YC-1 exposure time resulted in a decrease of the cell viability. Cell viability was measured by MTT assay. Data are presented as mean ± standard deviation from three independent experiments.

**Figure 3:**
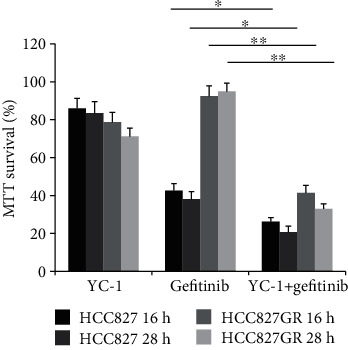
MTT assay in parental HCC827 cells and HCC827 GR cells with different treatments. Cell viability of cells with different treatments (blank control, YC-1, gefitinib, YC-1, and gefitinib combined, for 16 h and 28 h) was evaluated by MTT assay. The concentration of YC-1 was 40 *μ*M, and the final concentration of gefitinib was 20 nM. Error bars represented the mean ± standard deviation (SD). Data were obtained from three independent experiments. ^∗^*P* < 0.05 and ^∗∗^*P* < 0.01.

**Figure 4:**
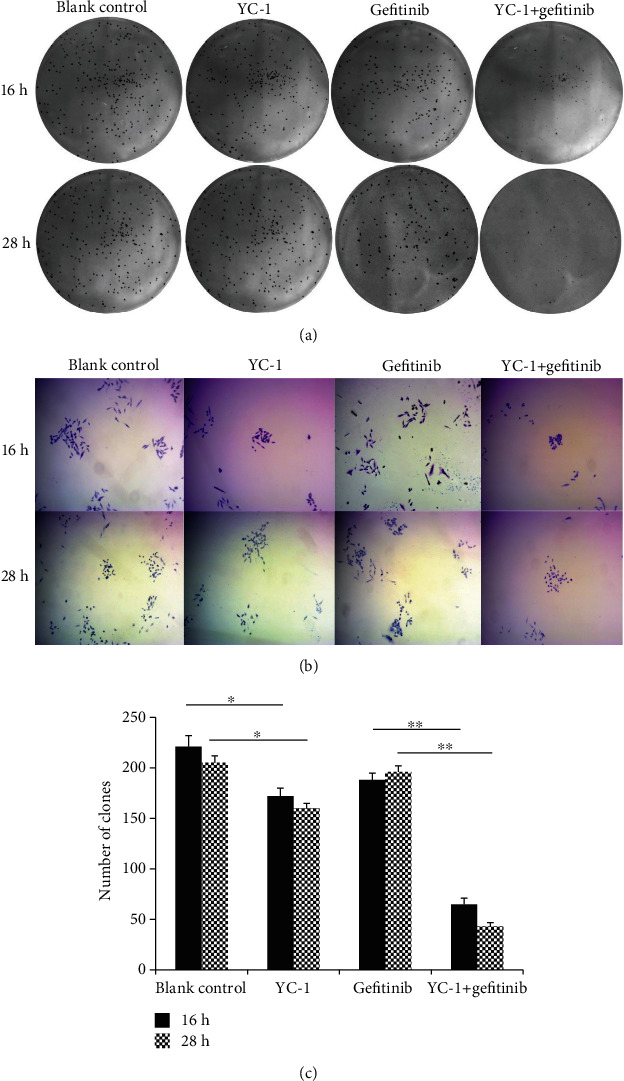
Colony formation analysis in HCC827 GR cells with different treatments. HCC827 GR cells were seeded and cultured on dishes with different treatments (blank control, YC-1, gefitinib, YC-1, and gefitinib combined, for 16 h and 28 h), then, cells were culture for 2 weeks in media without drugs. (a) Colony formation of HCC827 GR cells observed by naked eyes. (b) Colony formation of HCC827 GR cells observed under microscope (magnification, ×40). (c) Quantified results of colony formation analysis. The concentration of YC-1 was 40 *μ*M, and the final concentration of gefitinib was 20 nM. Error bars represented the mean ± SD. Data were obtained from three independent experiments. ^∗^*P* < 0.05 and ^∗∗^*P* < 0.01.

**Figure 5:**
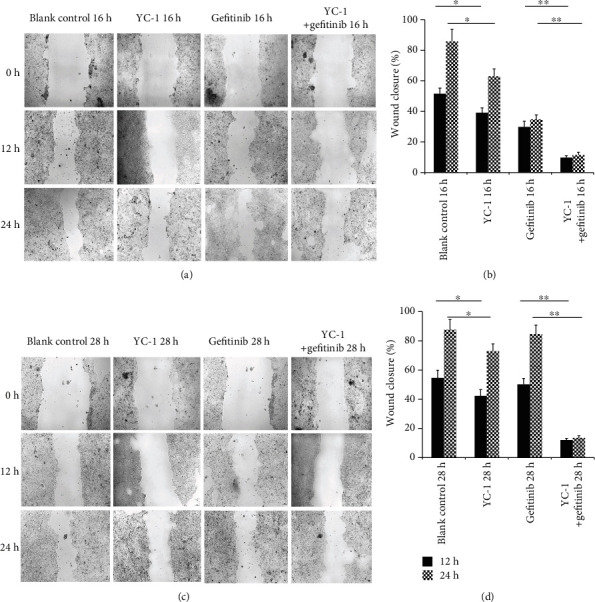
Wound healing assay in HCC827 GR cells with different treatments. HCC827 GR cells were pretreated with different treatments (blank control, YC-1, gefitinib, YC-1, and gefitinib combined, for 16 h and 28 h) before receiving the wound healing assay. (a) Wound healing status of HCC827 GR cells with different treatments for 16 h was presented after wounding for 12 and 24 h (magnification, ×100). (b) Wound-healing percentages after wounding for 12 and 24 h were calculated to evaluate cell migration ability of HCC827 GR cells with different treatments for 16 h. (c) Wound healing status of HCC827 GR cells with different treatments for 28 h was presented after wounding for 12 and 24 h (magnification, ×100). (d) Wound-healing percentages after wounding for 12 and 24 h were calculated to evaluate cell migration ability of HCC827 GR cells with different treatments for 28 h. The concentration of YC-1 was 40 *μ*M, and the final concentration of gefitinib was 20 nM. Error bars represented the mean ± SD. Data were obtained from three independent experiments. ^∗^*P* < 0.05 and ^∗∗^*P* < 0.01.

The concentration of YC-1 on HCC827 GR cells was determined through an MTT assay. The concentration of 40 *μ*M was finally chosen for this experiment, for a higher concentration of YC-1 was not able to further inhibit the viability of HCC827 GR cells (Figure 2(a)). Increase of the YC-1 exposure time resulted in a decrease of the cell viability, and the effect of 40 *μ*M YC-1 on HCC827 GR cells started at the time of 12 h and reached its optimum at the time of 24-28 h (Figure 2(b)). In order to avoid a false negative result caused by large groups of cell death while YC-1 and gefitinib combined, two time points of 16 h and 28 h were set for this study. Colony formation analysis, MTT assay, and wound healing assay were utilized to evaluate the sensitivity of HCC827 GR cells to gefitinib. In MTT assay, compared with gefitinib alone treated HCC827 GR cells, a reduction in cell viability was shown when HCC827 GR cells were treated with YC-1 and gefitinib combined for both 16 h and 28 h (*P* < 0.01; Figure 3), though this phenomenon was also presented in HCC827 cells (*P* < 0.05; Figure 3). In the colony formation analysis, YC-1 alone for both 16 h and 28 h can inhibit the colony formation ability of HCC827 GR cells (*P* < 0.05; Figure 4). Gefitinib and YC-1 together can also inhibit the colony formation ability of HCC827 GR cells (*P* < 0.01; Figure 4). In the wound healing assay, compared with gefitinib treatment alone, gefitinib and YC-1 combined treatment was able to inhibit cell migration (*P* < 0.01; Figure 5). YC-1 treatment alone for both 16 h and 28 h can also inhibit cell migration ability (*P* < 0.05; Figure 5).

### 3.3. Sensitivity of HCC827 Cells and HCC827 GR Cells to Gefitinib before and after Treatment with YC-1

**Figure 6:**
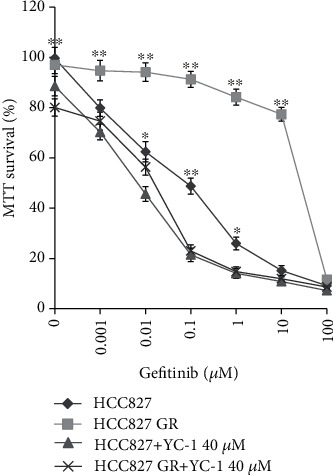
Comparison of gefitinib sensitivity among HCC827 cells, HCC827 GR cells, HCC827 cells treated with YC-1, and HCC827 GR cells treated with YC-1. Cell viability was measured by MTT assay. The concentration of YC-1 was 40 *μ*M. Data were obtained from three independent experiments and presented as mean ± SD. ^∗^*P* < 0.05 and ^∗∗^*P* < 0.01 for HCC827 cells and HCC827 GR cells versus those treated with YC-1.

HCC827 cells and HCC827 GR cells were treated with gefitinib at different concentrations (0.001, 0.01, 0.1, 1, 10, and 100 *μ*M) and 40 *μ*M YC-1 combined with gefitinib at different concentrations. Cell viability was measured by MTT. Compared with gefitinib alone-treated HCC827 cells, a reduction in cell viability was observed when HCC827 cells were treated with 40 *μ*M YC-1 and gefitinib at concentrations of 0.01, 0.1, and 1 *μ*M (*P* = 0.0348, *P* = 0.0085, and *P* = 0.01726, respectively). Compared with gefitinib alone-treated HCC827 GR cells, cell viability was reduced when HCC827 GR cells were treated with 40 *μ*M YC-1 and gefitinib at concentrations of 0, 0.001, 0.01, 0.1, 1, and 10 *μ*M (*P* = 0.0089, *P* = 0.0075, *P* = 0.00116, *P* < 0.001, *P* < 0.001, and *P* < 0.001, respectively). At the gefitinib concentration of 0.1 *μ*M, the sensitivity to gefitinib of HCC827 GR cells treated with 40 *μ*M YC-1 was enhanced compared with that of HCC827 cells treated with 40 *μ*M YC-1 (*P* = 0.0062). At other gefitinib concentrations, there was no significant difference at the sensitivity to gefitinib between HCC827 cells and HCC827 GR cells treated with 40 *μ*M YC-1 (Figure 6). These indicated that 40 *μ*M YC-1 was able to reverse the resistance of HCC827 GR cells to gefitinib and even presented enhanced sensitivity of HCC827 GR cells to gefitinib at treatment concentration compared with the parental cells.

### 3.4. The Detection of MET Amplification in HCC827 GR Cells and the Influence of HIF-1 Pathway Downregulation to MET Amplification

After the HCC827 GR cell line was established, MET amplification was detected. The MET level of HCC827 GR cells reached to more than 5 times of its parental cell. In the process of the HCC827 GR cell line established in a stepwise manner, the level of MET increased gradually. When the concentration of gefitinib reached to 1 *μ*M, a high level of MET amplification emerged.

**Figure 7:**
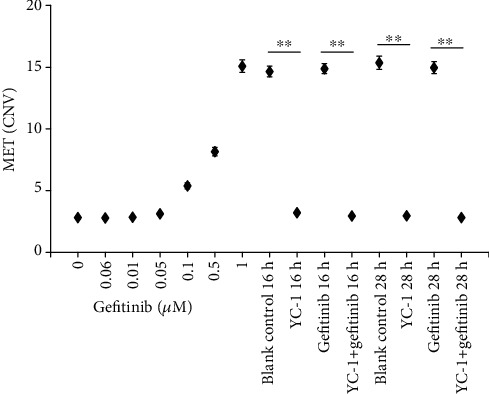
MET amplification detected by ddPCR CNV assay. In the process of the HCC827 GR cell line established, MET levels from parental HCC827 cell to HCC827 GR cell (the gefitinib concentration was increased gradually from 0 *μ*M to 1 *μ*M) were shown. MET levels of HCC827 GR cell with different treatment (blank control, YC-1, gefitinib, and YC-1 and gefitinib combined for 16 h and 28 h) were shown too. The concentration of YC-1 was 40 *μ*M, and the final concentration of gefitinib was 20 nM. Data were obtained from three independent experiments and presented as mean ± SD. ^∗∗^*P* < 0.01.

In different treatment groups of HCC827 GR, MET amplification was inhibited in the YC-1-treated group and YC-1 and gefitinib combined group for both 16 h and 28 h (*P* < 0.001 of both groups for 16 h and 28 h). It indicated that downregulation of the HIF-1 pathway was able to inhibit MET amplification (Figure 7).

### 3.5. Correlation between p-Met and HIF-1*α* Levels in HCC827 GR Cells with Different Treatments

**Figure 8:**
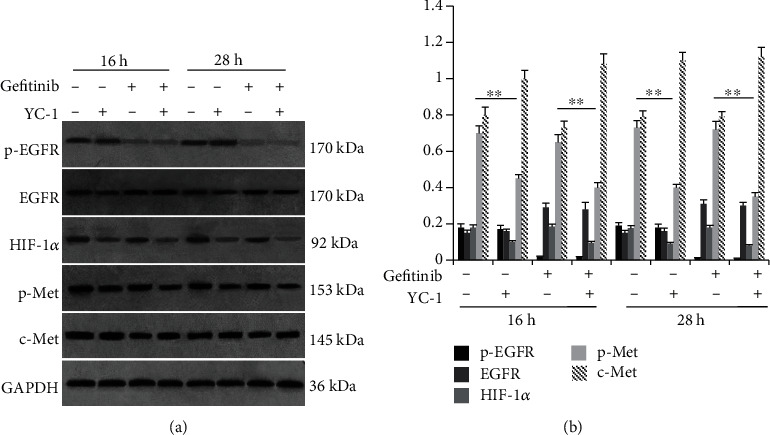
Expression of p-EGFR, EGFR, HIF-1*α*, p-Met, and c-Met in HCC827 GR cells with different treatments. (a) Western blot analysis was performed to detect p-EGFR, EGFR, HIF-1*α*, p-Met, and c-Met protein expression levels in HCC827 GR cells with different treatments (blank control, YC-1, gefitinib, YC-1, and gefitinib combined, for 16 h and 28 h). (b) Quantified densitometric scanning of the blots. The concentration of YC-1 was 40 *μ*M, and the final concentration of gefitinib was 20 nM. Error bars represented the mean ± SD. Data were obtained from three independent experiments. ^∗∗^*P* < 0.01.

**Figure 9:**
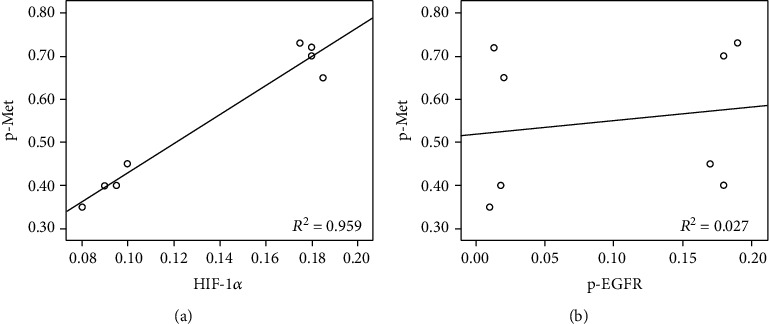
Pearson's correlation analysis between p-Met, HIF-1*α*, and p-EGFR levels. (a) There was a positive correlation between HIF-1*α* and p-Met levels in HCC827 GR cells with different treatments (*P* < 0.001; *R*^2^ = 0.959). (b) There was no correlation between p-Met and p-EGFR levels in HCC827 GR cells with different treatments (*P* = 0.697; *R*^2^ = 0.027).

Protein levels of p-EGFR, EGFR, HIF-1*α*, c-Met, and p-Met in different HCC827 GR groups were tested with western blot analysis. In the blank control group, the HIF-1*α* level and p-Met level were much higher than those in their parental HCC827 cells (*P* = 0.0029 and *P* < 0.001, respectively). In groups containing YC-1 treatment, levels of HIF-1*α* and p-Met were decreased compared with groups without YC-1 treatment (for HIF-1*α* levels comparison, *P* = 0.0359, *P* = 0.0125, *P* = 0.0297, and *P* = 0.0101, respectively; *P* < 0.001 for all p-Met levels comparison; Figure 8). In the above groups, the p-Met level was correlated with HIF-1*α* level (*P* < 0.001; *R*^2^ = 0.959; Figure 9(a)), but there was no correlation between the p-Met level and p-EGFR level (*P* = 0.697; *R*^2^ = 0.027; Figure 9(b)).

## 4. Discussion

In the present study, the inhibition of the HIF-1 pathway by YC-1 can make the HCC827 GR cell more sensitive to gefitinib. Through the comparison of gefitinib sensitivity among HCC827 cells, HCC827 GR cells, HCC827 cells treated with YC-1, and HCC827 GR cells treated with YC-1, it finally revealed that HIF-1 inhibitor YC-1 reversed the acquired resistance of HCC827 GR cells to gefitinib. In our previous research, we also found that the HIF-1 inhibitor was able to enhance the sensitivity of HCC827 cells to gefitinib [13].

Hypoxic tumor cells activate a series of signal pathways to adapt to the hypoxic condition. In these signal pathways, the HIF-1 signal pathway is the most well-defined and important pathway. HIF-1 pathway has more than 100 target genes which allow the tumor cells to survive and proliferate in hypoxic condition [20, 21]. HIF-1 keeps a stable construction in hypoxia. Then, it transfers to the nucleus and activates the expression of its target genes [8]. These activated genes prevent apoptosis and promote therapy resistance by regulating cell metabolism, survival, drug efflux, signaling, and DNA repair [22–26]. Thus, inhibiting the HIF-1 pathway is able to enhance the sensitivity of anticancer therapy theoretically.

For the HIF-1 pathway in EGFR-TKI therapy resistance, previous research showed the upregulation of HIF-1*α* [12]. Furthermore, the quantity of NSCLC stem cells which were resistant to EGFR-TKIs in EGFR mutant NSCLC was increased under hypoxic condition [11]. In acquired EGFR-TKI-resistant NSCLC cells with MET amplification, EGFR lost its regulation on MET, and whether the HIF-1 pathway remained the regulation on MET kept unclear [7]. Meanwhile, HCC827 GR is generated by exposing HCC827 cells to increasing concentrations of gefitinib, and MET amplification is the mechanism of its acquired resistance [16, 17]. In our study, MET amplification of HCC827 GR was presented by ddPCR CNV assay, and the correlations between HIF-1*α*, p-EGFR, and p-Met levels were analyzed by Pearson's correlation analysis. Our study showed that the p-Met level was correlated with the HIF-1*α* level, but there was no correlation between p-Met level and p-EGFR level. So, we speculated that the HIF-1 pathway keeps its regulation on MET while EGFR loses its regulation on MET in HCC827 GR cells with MET amplification. Accordingly, the regulation of the HIF-1 pathway on MET may be one of the mechanisms of YC-1 reversing the acquired resistance of HCC827 GR to gefitinib.

Despite the necessity of further researches to find out other possible mechanisms of YC-1 reversing the acquired resistance of HCC827 GR to gefitinib, the present study discovers that HIF-1 inhibitor YC-1 is able to reverse the acquired resistance of HCC827 GR with MET amplification to gefitinib. Therefore, the HIF-1 pathway may be a significant target for reversing the acquired resistance of NSCLC with MET amplification to EGFR-TKIs.

## 5. Conclusions

HIF-1 inhibitor YC-1 is able to reverse the acquired resistance of HCC827 GR to gefitinib, and the regulation of HIF-1 pathway on MET may be one of the mechanisms.

## Data Availability

The data used to support the findings of this study are available from the corresponding author upon request.

## Supplementary Materials

Supplementary Materials The supplementary material described methods of cell culture, medication treatment, western blot assay, MTT assay, colony formation assay, cell migration assay and statistical analyses.
