# Supplementary material for: HIF-1 Inhibitor YC-1 Reverses the Acquired Resistance of EGFR-Mutant HCC827 Cell Line with MET Amplification to Gefitinib
Source: Oxid Med Cell Longev. 2021 Mar 3;2021:6633867. doi: 10.1155/2021/6633867 (PMC7946473; doi:10.1155/2021/6633867)
Supplement: Supplementary Materials — The supplementary material described methods of cell culture, medication treatment, western blot assay, MTT assay, colony formation assay, cell migration assay and statistical analyses. [file 6633867.f1.docx]

Cell treatment

Previous study showed that the effect of YC-1 on HCC827 GR cells started at the time of 12 h and reached its optimum at the time of 24 h (1,2). In order to avoid a false negative result caused by large groups of cells death while YC-1 and gefitinib combined, two time points of 16 h and 28 h was set for this study. We also verified the time of effect starting by MTT assay before we design this study.

Cells were separated into four groups to examine the effect of HIF-1 inhibitor YC-1 on the sensitivity of HCC827 GR cells to gefitinib: A blank control group, a YC-1 group, a gefitinib group, a YC-1 and gefitinib group. For the blank control group, the HCC827 cells were cultured for 16 and 28 h (37 ˚C and 5% CO2) in original medium. For the YC-1 group, HCC827 cells were cultured for 16 and 28 h (37 ˚C and 5% CO2) in medium with 40 μM YC-1. For the gefitinib group, following the culturing of HCC827 cells in its original culture for 4 h, gefitinib was added into the medium at a final concentration of 20 nM and treated for 16 and 28 h (37 ˚C and 5% CO2). For the YC-1 and gefitinib combined group, subsequent to the culture of HCC827 cells in medium with 40 μM YC-1 for 4 h, gefitinib was added into the existing medium at a final concentration of 20 nM and treated for 16 and 28 h (37 ˚C and 5% CO2).

Western blot assay.

Cells treated with the aforementioned different treatments were digested and collected for total protein extraction and treated with a lysis buffer containing 20 mmol/l Tris (PH 7.5), 150 mmol/l NaCl, 1% Triton X-100 and inhibitors of protease and phosphates on ice for 30 min. The cell lysis products were centrifuged for 15 min at 12000 x g in a 4˚C refrigerated centrifuge and the supernatants were collected. The final protein concentration was measured using a BCA protein kit according to the manufacturer’s protocol (Thermo Fisher Scientific, Inc., Waltham, MA, USA) and supernatants were boiled for 5 min. Next, 100 μg protein lysates were separated using 12% SDS-PAGE. The proteins were transferred to polyvinylidene fluoride membranes and the membranes were blocked with 5% skimmed milk with Tris-buffered saline (TBS) and Tween 20 (TBST) at room temperature for 2 h. A total of 137 mM NaCl, 20 mM Tris and 0.05% Tween 20 were contained in TBST of which the PH was adjusted with HCl to pH7.5. The blotted membrane was incubated with primary antibodies against p-Met (1:500), p-EGFR (1:500), HIF-1α (1:500) and GAPDH ((cat. no. ab9484; 1:1000; Abcam, Cambridge, MA, USA) at room temperature for 2 h. The membrane was washed with TBST three times and then incubated with horseradish peroxidase‑-conjugated goat anti-rabbit immunoglobulin G secondary antibody (cat. no. RABHRP1-10UL; 1:1000; Sigma-Aldrich; Merck KGaA) at room temperature for 1.5 h. The immunoreactive bands were washed with TBST four times and observed using enhanced chemiluminescence (ECL) plus detection reagent (Pierce, Rockford, IL, USA). GAPDH was used as an internal control. The densitometry of the bands was quantified by UVP Gel Imaging System Labworks 4.6 software (LabWorks; UVP, LLC, Phoenix, AZ, USA).

MTT assay.

HCC827GR cells were seeded at a density of 20,000 cells per well in 96-well plates and maintained in RPMI-1640 medium supplemented with 10% FBS and 1% penicillin-streptomycin. Following overnight incubation (37˚C and 5% CO2), cells were exposed to different treatments (blank control, YC-1, gefitinib, and YC-1 and gefitinib combined for 16 and 28 h). Following treatments, the MTT reagent (Sigma-Aldrich; Merck KGaA) was added and cells were incubated at 37˚C for 4 h. Subsequently the medium is removed and 150 μl dimethyl sulphoxide (DMSO) is added to dissolve the purple formazan salt crystals. Then optical density was measured on a microplate reader at a wavelength of 490nm.

Colony formation assay.

Cells with different treatments as aforementioned were seeded onto culture plates. Cells were seeded at low density (300 cells per plate) and cultured with different treatments as aforementioned. Then cells were cultured in RPMI-1640 medium supplemented with 10% FBS and 1% penicillin-streptomycin for 2 weeks, in a humidified atmosphere with 5% CO2 at 37˚C. Then, Colonies were stained with 0.05% crystal violet solution for 20 min at room temperature. Finally, the number of colonies with >10 cells were counted under an invert microscope (×10).

Cell migration assay.

Subsequent to the aforementioned treatments, HCC827GR cells were plated into 6-well plates and cultured under serum starvation to a maximum of 60% confluence. A scratch was produced 16 h after the beginning of serum starvation of the cells. Each well was wounded by scratching with a 10μl pipette tip, following PBS washes for three times to remove cell debris. The gap distance of the wound was measured at three different sites using Image J Software (v. 1.48q; National Institutes of Health, Bethesda, MD, USA) in pixels. Wound closure was observed at 0, 24 and 48 h after wound simulation. Graphs were plotted against the percentage of the migration distance that the cells moved.

Reference

1. Yeo EJ, Chun YS, Cho YS, Kim J, Lee JC, Kim MS and Park JW: YC-1: a potential anticancer drug targeting hypoxia-inducible factor 1. J Natl Cancer Inst 95(7): 516-525, 2003.
2. Tsui L, Fong TH and Wang IJ: YC-1 targeting of hypoxia-inducible factor-1alpha reduces RGC-5 cell viability and inhibits cell proliferation. Mol Vis 18:1594-1603, 2012.
